# Latent transition analysis of stigma and its association with treatment adherence in pulmonary tuberculosis patients during anti-tuberculosis therapy

**DOI:** 10.3389/fpubh.2026.1797748

**Published:** 2026-04-16

**Authors:** Shuyin Qian, Xiangyun Wang, Bei Huang, Haiyan Lu, Cuicui Xu, Juan Shen

**Affiliations:** Sixth People's Hospital of Nantong, Nantong, China

**Keywords:** latent profile analysis, latent transition analysis, pulmonary tuberculosis, stigma, treatment adherence

## Abstract

**Objective:**

To explore the changes in latent categories of stigma among patients with pulmonary tuberculosis over time and their correlation with treatment adherence.

**Methods:**

A total of 236 patients with pulmonary tuberculosis were selected as the research subjects using the convenience sampling method. The social impact scale and medication adherence report scale were used to measure the enrolled research subjects at 1 month (T1) and 3 months (T2) after standard treatment. Latent profile analysis, latent transition analysis, and logistic regression were employed for statistical analysis.

**Results:**

The scores of treatment adherence of pulmonary tuberculosis patients at T1 were significantly different according to marital status (χ^2^^2^ = 11.089, *P* = 0.001), education background (χ^2^ = 19.4027, *P* < 0.001), and living style (χ^2^ = 6.631, *P* = 0.010). At T2, the scores of treatment adherence were significantly different according to age (χ^2^ = 9.485, *P* = 0.002), marital status (χ^2^ = 9.567, *P* = 0.002), education (χ^2^ = 10.604, *P* = 0.005), and living style (χ^2^ = 6.186, *P* = 0.013). At T1 and T2, there were two latent profile categories of stigma, namely the high stigma group (C1) and the low stigma group (C2). The results of latent transition analysis showed that 38.65% of patients with pulmonary tuberculosis were continuously in the C1 group (continuous high stigma group), 28.37% of patients with pulmonary tuberculosis were continuously in the C2 group (continuous low stigma group), 24.16% of patients with pulmonary tuberculosis changed from the C1 group to the C2 group (decreasing stigma group), and 8.82% of patients with pulmonary tuberculosis changed from the C2 group to the C1 group (increasing stigma group). In the multivariate Logistic regression analysis, compared with the continuous low stigma group, the risk of low treatment adherence of pulmonary tuberculosis patients in the continuous high stigma group (OR = 10.740), the decreasing stigma group (OR = 2.145), and the increasing stigma group (OR = 4.166) increased (all *P* < 0.001).

**Conclusions:**

This study found that there were four dynamic trajectories of stigma in patients with pulmonary tuberculosis: continuous high stigma group (38.65%), continuous low stigma group (28.37%), decreasing stigma group (24.16%), and increasing stigma group (8.82%). Compared with the continuous low stigma group, the risk of low treatment adherence of patients in the other three groups was significantly increased.

## Introduction

As a major global public health challenge, the effectiveness of tuberculosis prevention and control depends not only on the progress of diagnosis and treatment technology but also on patients' treatment adherence (1). The data show that there will be an estimated 10.7 million new TB cases worldwide in 2024, of which 696,000 will be diagnosed in China. The disease burden is still serious, especially the problem of drug resistant TB, which is difficult to treat and imposes a significant burden (2, 3). The prevention and control of tuberculosis not only depends on the progress of medical technology but also requires more attention to be paid to patients' treatment adherence. Patients' adherence to treatment has become a key link affecting the overall prevention and control effect (4, 5).

As an important psychosocial factor, stigma is increasingly regarded as the core mechanism of treatment adherence. Stigma refers to the complex negative emotional experiences of patients (6), such as shame, inferiority, and guilt, caused by their own diseases. Studies have shown that more than 60% of patients have experienced a moderate to high level of stigma, which can be traced back to the historical stigmatizing label of the disease, social fear and misunderstanding of infectious diseases, and the self denial tendency of patients (7, 8). This negative psychological experience not only significantly affects patients but also leads to a higher comorbidity rate of depression is more directly to weaken the patient's medical behavior (9, 10). These factors include reduced confidence in the efficacy of treatment, resulting in patients concealing their illness, interrupting medication, or avoiding follow up (11).

However, current studies in this field are mostly confined to cross-sectional designs. These designs can only reveal the static correlation between stigma and treatment adherence at a specific point in time, and it is challenging to capture the dynamic interaction and latent causal path between the two during the anti-tuberculosis treatment process (12, 13). Cross-sectional studies are unable to effectively identify the evolution trajectory of patients' stigma across different treatment stages. Moreover, their capacity to control time confounding factors is limited, which restricts the precise formulation of intervention strategies (14, 15).

Latent transition analysis is used to study the individual latent categories of ownership transformation at different time points in longitudinal statistical methods. Its core advantage lies in revealing the dynamic change law of population heterogeneity through transition probability (16). Based on longitudinal follow up data, it can identify different latent categories of shame among patients with tuberculosis. Accurately quantifying the transition probability of different categories between different treatment stages helps to reveal the key psychosocial turning points that affect the change of adherence points (17).

In view of this, this study used latent profile analysis to divide the stigma of patients with pulmonary tuberculosis into multiple mutually exclusive latent subgroups, so as to reveal the heterogeneity of patients' stigma. Latent transition analysis was used to evaluate the development of stigma in different subgroups after 1 month and 3 months of standardized treatment for pulmonary tuberculosis over time. The transition probability was used to reflect the phased development law of stigma in patients with pulmonary tuberculosis. And from a dynamic perspective, it probes into the influence of the change of disease related shame on medical behavior, providing new ideas for increasing the medical compliance of tuberculosis patients in subsequent treatment.

## Research objects and methods

### Subjects

From July 2024 to June 2025, a total of 236 patients with pulmonary tuberculosis admitted to the Sixth People's Hospital of Nantong, Jiangsu Province, were continuously included as the research objects using the convenience sampling method.

Inclusion criteria:

1. Meeting the diagnostic criteria of “Guidelines for the Diagnosis and Treatment of Pulmonary Tuberculosis” (18);
2. Age ≥ 18 years;
3. Having basic communication skills and having signed the informed consent.

Exclusion criteria:

1. Secondary pulmonary tuberculosis or drug resistant pulmonary tuberculosis;
2. Patients with mental or psychological diseases or symptoms;
3. Failure to complete two measurements.

This study has been approved by the Sixth People's Hospital of Nantong, Jiangsu Province (approval number: 2024 07 033) and meets the ethical requirements. All subjects voluntarily participated in this study and signed the informed consent form.

### Sample size calculation

Follow the repeated measures sample size formula (19)


$$n=\frac{2}{\delta^{2}}[\sigma_{\mu }^{2}+\frac{1+(K-1)\rho_{c}}{K}{\sigma_{e}}^{2}]{\left(U_{\alpha /2}+U_{\beta }\right)}^{2}$$

Combined with the pilot study, the minimum sample size was determined as *n* = 192÷ (1%−10%) =214, considering the 10% dropout rate of longitudinal investigation.

### Survey tools

#### General information questionnaire

The general information questionnaire included general demographic data (age, gender, marital status, parental status, place of residence, living style, monthly income), Body Mass Index (BMI), and complications of other diseases among patients with pulmonary tuberculosis.

### Social impact scale (SIS)

Developed by Fife et al. (20), this study utilizes the Chinese version of the Social Impact Scale by Huang Chaojun et al. (21), and has been applied to measure the level of stigma in patients with a variety of diseases. The scale contains four dimensions, namely economic discrimination (six items), social exclusion (six items), social isolation (six items), and intrinsic shame (six items). The scores for “always” and “never” are assigned from 1 to 4 respectively, and the total score ranges from 24 to 96. The higher the score, the higher the level of stigma. The Cronbach's α coefficient of the total scale is 0.908, the content validity is 0.944, and the correlation coefficient of each item is 0.457 0.783 (*P* < 0.01). In this study, the Cronbach's α coefficients of the scale are 0.844 and 0.890.

### Medication adherence report scale (MARS)

This study used the Chinese version of the medication adherence scale (Yin Xiaoxu, unpublished)^1^, developed by Yin Xiaoxu, to measure the treatment adherence of patients with pulmonary tuberculosis. The scale consists of 10 items, including medication adherence behavior, medication attitude, the negative impact of adverse reactions, and attitude toward psychotropic drugs. Each item has two options: “yes” and “no”, and the total score ranges from 0 to 10. A score greater >than 6 indicates good adherence^1^. The Cronbach's α coefficient, test retest reliability, and split half reliability of the scale are 0.87, 0.83, and 0.85, respectively. Pearson correlation analysis shows that the total score of the scale is significantly correlated with the scores of each dimension (*P* < 0.001), suggesting that the scale has good content validity. The Cronbach's α coefficients of the two measurements of the scale in this study are 0.897 and 0.905.

### Data collection methods

Before conducting the survey, all researchers participating in the survey were trained. After obtaining the consent of hospitals and patients, a questionnaire survey was carried out, and the general information questionnaire was retrieved from the patients' medical records. The standard course of treatment for newly diagnosed pulmonary tuberculosis is 6 months. The specific treatment plan is divided into two stages: a 2 month intensive period and a 4 month consolidation period.

To understand the stigma level and treatment adherence of patients in the two stages, the stigma scale and treatment adherence assessment scale were collected at the 1 month (T1) and 3 month (T2) time nodes after standard treatment. Throughout the study, patients' privacy was strictly protected, and all surveys were completed in an independent and confidential environment. All surveys were conducted by the researchers via the out of hospital follow up system. The questionnaires were filled out anonymously, and patients were clearly promised that all data would be used solely for the purpose of this study.

To ensure data quality, all investigators were trained, the requirements for filling out the questionnaire and the data collection process were standardized, and leading questions were prohibited.

### Statistical methods

SPSS 26.0 software was used for descriptive statistics and correlation analysis. Enumeration data were presented as the number of cases and percentage. Measurement data that followed a normal distribution were expressed as mean ± standard deviation. The chi-square test was used to compare different general demographic characteristics, and the Harman single factor test was used for the common method deviation test. Mplus 8.3 software was used for latent profile analysis and latent transition analysis, and model fit test indicators were compared. The model fit tests included the Akaike information criterion (AIC), Bayesian information criterion ((BIC), sample-corrected BIC (aBIC), Entropy, Lo-Mendell-Rubin adjusted likelihood ratio test (LMR), and bootstrap-based likelihood ratio test (BLRT). The smaller the AIC, BIC, and aBIC results, the better the model fit. The value of Entropy ranges between 0 and 1, and generally, Entropy >0.8 is required for high explanatory power. Univariate logistic regression was used to analyze the relationship between the stigma transformation mode and treatment adherence, and multivariate stepwise regression was used to control the confounding effect. A *p*-value < 0.05 was considered statistically significant.

## Results

### General information of respondents

During the follow up period, 13 patients withdrew due to accidental death or refusal to answer the questions. A total of 223 patients completed two follow up surveys. A comparison of the general characteristics between the dropout group (*n* = 13) and the final participants (*n* = 223) showed no statistically significant differences (*P* > 0.05). Basic information of the participants is provided in Table 1.

**Table 1:** General Information of the respondents (*n* = 223).

Items	Categories	*N*	Percentage (%)
Age (years)	< 60	100	44.84
	≥60	113	50.67
Gender	Male	160	71.75
	Female	63	28.25
Marital status	Unmarried/divorced/widowed	26	11.66
	Married	197	88.34
Status of children	Having children	185	82.96
	No children	38	17.04
Educational level	Junior high school or below	94	42.15
	High school	93	41.70
	College and above	36	16.14
Place of residence	City	105	47.09
	Township/rural	118	52.91
Style of residence	Living alone	12	5.38
	Live together with family	211	94.62
Monthly income (yuan)	< 3,000	41	18.39
	3,000 ~ 6,000	77	34.53
	6,001 ~ 10,000	80	35.87
	>10,000	25	11.21
BMI (kg/cm^2^)	< 18.5	50	22.42
	18.5 ~ 23.9	142	63.68
	>24	31	13.90
Hypertension	yes	62	27.80
	No	161	72.20
Diabetes	yes	50	22.42
	No	173	77.58
Coronary heart disease	Yes	57	25.56
	No	166	74.44

### Common method deviation test

This study found that in the common methods bias test for longitudinal survey data, the samples collected at two time points, T1 and T2, were separately tested using the Harman single factor test. The results showed that the number of factors with eigenvalues greater than 1 in the T1 and T2 data was 11 and 10, respectively, and the proportion of variance explained by the first common factor was 15.77 and 19.48%, respectively, which were lower than the critical value standard of 40% (22). This indicates that the data in this study were not affected by serious common method bias.

### Longitudinal measurement invariance test

To ensure that the “true change” observed in the latent transition analysis was not caused by differences in the meaning of the measurement instrument across time points, this study first tested longitudinal measurement invariance.

We constructed a configural invariance model and a strong invariance model. The configural invariance model only required the factor structure to be the same at T1 and T2; the strong invariance model further constrained the factor loadings and intercepts (or thresholds) to be equal across the two time points.

Model fit results showed that, compared to the configural invariance model, the change in fit indices for the strong invariance model (ΔCFI = 0.01, ΔRMSEA = 0.005) was below the commonly accepted cutoff values. This indicates that the categories had the same meaning at T1 and T2.

### Comparison of treatment adherence scores of pulmonary tuberculosis patients at T1 and T2

Marital status (χ^2^ = 11.089, *P* = 0.001), educational background (χ^2^ = 19.407, *P* < 0.001), and living style (χ^2^ = 6.631, *P* = 0.010) were significantly different in the treatment adherence scores of tuberculosis patients at T1. The treatment adherence scores at T2 were significantly different in age (χ^2^ = 9.485, *P* = 0.002), marital status (χ^2^ = 9.567, *P* = 0.002), education (χ^2^ = 10.604, *P* = 0.005), and living style (χ^2^ = 6.186, *P* = 0.013), as shown in Table 2.

**Table 2:** Comparison of treatment adherence scores of tuberculosis patients at T1 and T2 (*n* = 223).

Variables	T1 treatment adherence score		χ^2^	*P*-value	T2 treatment adherence score		χ^2^	*P*-value
	**0** ~**6**	>**6**			**0** ~**6**	>**6**		
Age (years)								
< 60	28	72	1.846	0.131	37	63	9.485	0.002
≥60	45	78			71	52		
Gender								
Male	52	108	0.014	0.905	78	82	0.023	0.879
Female	21	42			30	33		
Marital status								
Unmarried/divorced/widowed	16	10	11.089	0.001	20	6	9.567	0.002
Married	57	140			88	109		
Education								
Junior high school and below	46	48	19.407	< 0.001	60	44	10.604	0.005
High school	19	74			37	56		
Junior college and above	8	28			11	25		
Place of residence								
City	29	76	2.359	0.125	47	58	1.069	0.301
Countryside	44	74			61	57		
Lifestyle								
Living alone	8	4	6.631	0.010	10	2	6.186	0.013
Live together with family	65	146			98	113		
Monthly household income (yuan)								
< 4000	15	26	1.484	0.686	21	20	0.219	0.974
4000 ~ 6999	22	55			39	38		
7000 ~ 10000	26	54			38	42		
>10,000	10	15			10	10		
BMI (kg/cm^2^)								
< 18.5	11	39	4.864	0.088	24	26	0.146	0.930
18.5 ~ 23.9	48	94			68	74		
>24	14	17			16	15		
Hypertension								
Yes	22	40	0.295	0.587	35	27	2.212	0.137
No	51	110			73	88		
Diabetes								
Yes	17	33	0.047	0.829	28	22	1.479	0.224
No	56	117			80	93		
Coronary heart disease								
Yes	19	38	0.012	0.911	33	24	2.746	0.097
No	54	112			75	91		

### Latent profile analysis of stigma in patients with pulmonary tuberculosis

**Figure 1:**
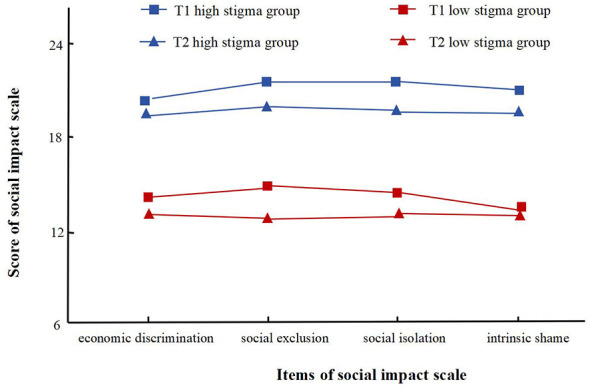
Line chart of latent profile analysis of stigma in patients with pulmonary tuberculosis.

Based on the scores of the four dimensions of the stigma scale, this study used latent profile analysis to evaluate the patterns of stigma manifestation in patients. Starting from the baseline model (single class), models with 1–4 latent classes were fitted successively, and the corresponding fit indices are presented in Table 3. At both T1 and T2 time points, the model fit results showed that, under the condition that both the Likelihood Ratio Test (LRT) and the Bootstrap Likelihood Ratio Test (BLRT) reached statistical significance, smaller values of AIC, BIC, and aBIC indicated better model fit. Additionally, an entropy value greater than 0.8 suggested good precision and reliability of classification. Therefore, the 2-class latent class model was ultimately determined to be optimal. Based on the characteristics of each class in terms of the total stigma score, class 1 (higher score) was named the high stigma group, and class 2 (lower score) was named the low stigma group. The specific distribution is shown in Figure 1.

**Table 3:** Comparison of fitting parameter indexes of different latent profile models.

Time	Model	AIC	BIC	aBIC	Entropy	LRT	BLRT	Class probability
T1	1	3304.952	3327.812	3316.541	–	–	–	–
	2	3155.785	3183.916	3182.134	0.862	0.004	< 0.001	0.60/0.40
	3	3184.133	3203.830	3197.217	0.840	0.068	< 0.001	0.23/0.47/0.30
	4	3204.074	3228.242	3209.910	0.853	0.105	0.189	0.26/0.11/0.25/0.38
T2	1	3365.287	3396.258	3386.863	–	–	–	–
	2	3099.933	3113.294	3105.921	0.876	0.000	< 0.001	0.45/0.55
	3	3227.987	3241.740	3240.815	0.822	0.059	< 0.001	0.28/0.47/0.25
	4	3162.558	3195.842	3185.846	0.865	0.113	< 0.001	0.20/0.18/0.27/0.35

### Latent transition analysis of stigma in patients with pulmonary tuberculosis

Latent transition analysis was used to explore the transformation of the latent profile of stigma in patients with pulmonary tuberculosis at T1 and T2 time points. The results showed that 67.02% of patients with pulmonary tuberculosis had the probability of maintaining their original latent state. Specifically, 38.65% of the patients with pulmonary tuberculosis remained continuously in the high stigma group, so they were named the continuous high stigma group; 28.37% of the patients with pulmonary tuberculosis stayed continuously in the low - stigma group, and thus they were named the continuous low stigma group; 24.16% of the patients with pulmonary tuberculosis shifted from the high stigma group to the low stigma group, and they were named the decreased stigma group. Moreover, 8.82% of the tuberculosis patients changed from the low stigma group to the high stigma group and were named the rising - stigma group, as shown in Table 4.

**Table 4:** T1-T2 transition probability of stigma in patients with tuberculosis (*n*=223).

	Items	T2	
		C1: high stigma group	C2: low stigma group
T1	C1: high stigma group	38.65	24.16
	C2: low stigma group	8.82	28.37

### The impact of stigma transition patterns on treatment adherence in patients with pulmonary tuberculosis

Furthermore, the influence of the stigma transformation mode of pulmonary tuberculosis patients on treatment adherence was analyzed. Taking the continuous low stigma group as the reference group, univariate and multivariate regression analyses were carried out. With the stigma transformation mode of pulmonary tuberculosis patients as the independent variable and treatment adherence (>6 = 0, 0 ~ 6 = 1) as the dependent variable, in the univariate Logistic regression analysis, compared with the continuous low stigma group, pulmonary tuberculosis patients in the continuous high stigma group, the decreasing stigma group, and the increasing stigma group had an increased risk of low treatment adherence (all *P* < 0.001). To more accurately assess the independent effect of latent classes on treatment adherence at T2, this study included the adherence score at T1 as a covariate in Model 3 for baseline adjustment. This strategy aimed to control for individual differences at baseline, thereby isolating the net effect of stigma classes on changes in adherence. To address potential multicollinearity, the variance inflation factor (VIF) was calculated. The results showed that the VIF for all variables was less than 5, indicating no severe multicollinearity and stable parameter estimation.

In Model 2 (controlling for general demographic characteristics) and Model 3 (controlling for general demographic characteristics and T1 treatment adherence), the risk of low treatment adherence increased in pulmonary tuberculosis patients in the decreasing stigma group and the increasing stigma group (all *P* < 0.05). However, the risk was the lowest in the decreasing stigma group (OR = 1.654), as shown in Table 5.

**Table 5:** The association between stigma and treatment adherence in patients with pulmonary tuberculosis.

Change patterns	*B*	*SE*	OR	95%CI	*P*-value
Model 1					
Sustained low stigma group	–	–	–	–	–
Continuous high stigma group	3.302	0.533	27.167	9.557 ~ 77.222	< 0.001
Decreased stigma group	0.960	0.315	2.612	1.409 ~ 4.842	0.001
Increased stigma group	1.914	0.339	6.780	3.489 ~ 13.177	< 0.001
Model 2					
Continuous low stigma group	–	–	–	–	–
Continuous high stigma group	2.925	0.517	18.634	6.764 ~ 51.332	< 0.001
Decreased stigma group	0.904	0.328	2.469	1.298 ~ 4.697	0.008
Rising stigma group	1.698	0.467	5.463	2.187 ~ 13.644	< 0.001
Model 3					
Sustained low stigma group	–	–	–	–	–
Continuous high stigma group	2.374	0.479	10.740	4.200 ~ 27.463	< 0.001
Decreased stigma group	0.763	0.320	2.145	1.145 ~ 4.016	0.027
Rising stigma group	1.415	0.413	4.116	1.832 ~ 9.249	< 0.001

Model 1: Logistic regression; Model 2: controlling for age, gender, marital and children status, place of residence, mode of residence, monthly income), body mass index, and concurrent diseases; Model 3: controlling age, gender, marital and children status, residence, living style, monthly income), body mass index, concurrent diseases and treatment adherence at T1 time.

## Discussion

Hafez et al. pointed out that treatment adherence among tuberculosis patients is the key to curing the disease, preventing drug resistance, and avoiding infecting others, which is directly related to treatment outcomes and life safety (23). The results of this study showed that the treatment adherence of patients with pulmonary tuberculosis was significantly affected by demographic characteristics and social support factors at different stages of treatment. After 1 month of standardized treatment, marital status, educational background, and living style had a significant impact on treatment adherence. Married patients usually receive financial and emotional support from their spouses and are more likely to adhere to medication during treatment (24). Patients with a higher education level have a more comprehensive understanding of the disease and a better grasp of the treatment plan (25). Patients living with their families can receive more comprehensive daily care and medication supervision, and these factors together contribute to the improvement of treatment adherence. After 3 months of standardized treatment, age, marital status, educational background, and living style had a significant impact on treatment adherence. Older patients may bear a heavier psychological burden due to a longer disease course and a decline in physical function (26). Unmarried or divorced patients lack a family support system and are more likely to experience low self - esteem and shame. Patients with a low educational background have insufficient knowledge of the disease and are prone to misunderstandings and negative emotions. Patients living alone lack emotional release channels, and their treatment adherence is poorer (27).

This suggests that clinical staff should focus on patients who are older adult, have a low education level, live alone, or are unmarried, strengthen health education and psychological counseling, and improve patients' correct understanding of the disease. At the same time, the family support system should be actively mobilized. Family members should be encouraged to participate in the treatment process, and treatment adherence can be improved through spousal medication supervision. In addition, the psychological state of patients should be evaluated regularly, negative emotions should be detected and intervened in a timely manner, and the treatment adherence and quality of life of patients should be improved through psychological interventions such as cognitive - behavioral therapy and mindfulness - based stress reduction.

The results of this study show that the stigma experienced by patients with pulmonary tuberculosis exhibits relatively stable characteristics during treatment, but there are dynamic changes in some patients. 67.02% of the patients remained in the original latent state, indicating that the stigma has a certain degree of stability, which may be related to the relatively fixed perception of the disease, the social support system, and the psychological coping style of patients. 38.65% of the patients continued to be in the high - stigma group, suggesting that these patients may face a long - term psychological burden. The reasons may include excessive worry about disease infectivity, persistent social discrimination, lack of effective psychological counseling, and insufficient family support. 28.37% of the patients continued to be in the low - stigma group, indicating that these patients can adapt well to the disease, which may be related to good disease awareness, positive psychological adjustment ability, and adequate social support (28). 24.16% of the patients changed from the high - stigma group to the low - stigma group, indicating that through standardized treatment and effective psychological intervention, the patients' stigma can be significantly alleviated, which may be due to the relief of symptoms during treatment, health education and psychological support provided by medical staff, and the gradual improvement of social perception of the disease (29). However, 8.82% of the patients changed from the low - stigma group to the high - stigma group, suggesting that some patients may experience a deterioration of their psychological state during treatment, which may include the occurrence of treatment side - effects, the continuous impact of social discrimination, an increased economic burden, or problems in the family support system (30, 31).

Medical staff should pay attention to the stigma assessment of pulmonary tuberculosis patients, identify patients with high stigma in the early stage of treatment, and implement early psychological intervention. For patients with persistent high stigma, individualized psychological support and cognitive - behavioral intervention should be provided. At the same time, attention should be paid to patients with increasing stigma during the treatment process, and relevant risk factors should be identified and intervened in a timely manner. In addition, social publicity should be strengthened to reduce discrimination against patients with pulmonary tuberculosis, and a good social support environment should be created to promote the psychological rehabilitation of patients.

The results of this study show that there is a significant negative correlation between the stigma and treatment adherence of patients with pulmonary tuberculosis. That is, the higher the level of stigma, the greater the risk of low treatment adherence among patients. The patients in the continuous high - stigma group (38.65%) had poor treatment adherence due to long - term stigma, possibly because of the wrong perception of the disease, the continuous influence of social discrimination, and the lack of effective psychological counseling. In the decreased - stigma group (24.16%), although the stigma was alleviated, there was still a certain degree of psychological burden, which affected treatment adherence. Patients with increasing stigma (8.82%) may experience psychological deterioration during treatment, such as the occurrence of treatment side - effects, an increased economic burden, or problems in the family support system, which further reduce treatment adherence (32).

The clinical implication is that medical staff should pay attention to the assessment of stigma in patients with pulmonary tuberculosis, identify patients with high stigma in the early stage of treatment, and implement early psychological intervention. For patients with continuous high stigma, individualized psychological support and cognitive - behavioral intervention should be provided. At the same time, attention should be paid to patients with increasing stigma during the treatment process, and relevant risk factors should be identified and intervened in a timely manner. In addition, social publicity should be strengthened to reduce discrimination against patients with pulmonary tuberculosis, and a good social support environment should be created to promote the psychological rehabilitation of patients.

### Limitations

This study has some limitations. The convenience sampling method employed at a single center may diminish the representativeness and universality of the sample. Secondly, the extensive survey period may heighten the risk of a decline in quality control. In the future, the results will be optimized in light of the aforementioned problems. Simultaneously, we will concentrate on formulating and implementing intervention measures to address the psychological issues of patients following pulmonary tuberculosis surgery, thereby facilitating patients' better reintegration into society and daily life. Additionally, data on both stigma and treatment adherence were collected via patient self-report scales within the same follow-up system, which may lead to common method bias (CMB). Although we conducted a preliminary Harman's single-factor test, the results did not indicate that a single factor explained most of the variance. However, we acknowledge that this method has limited power in excluding potential biases such as social desirability or common rater effects. To partially mitigate this risk, we controlled for sociodemographic variables (e.g., age, gender, marital status) in subsequent regression analyses to reduce systematic bias. Furthermore, as stigma is a subjective psychological construct, its measurement inherently relies on patient self-report, which to some extent constrains the possibility of triangulation with objective indicators. Future studies could consider incorporating objective clinical measures (e.g., blood drug concentration monitoring) or employing multi-timepoint, multi-source data collection methods to further validate the robustness of the findings in this study.

## Conclusion

38.65% of the patients with pulmonary tuberculosis remained continuously in the high stigma group, 28.37% of the patients with pulmonary tuberculosis remained continuously in the low stigma group, 24.16% of the patients with pulmonary tuberculosis shifted from the high stigma group to the low stigma group, and 8.82% of the patients with pulmonary tuberculosis shifted from the low stigma group to the high stigma group. Compared with the continuous low stigma group, the pulmonary tuberculosis patients in the continuous high stigma group, the decreasing stigma group, and the increasing stigma group faced an increased risk of low treatment adherence.

## Data Availability

The original contributions presented in the study are included in the article/supplementary material, further inquiries can be directed to the corresponding author.

## Footnotes

1 Yin Xiaoxu. Research on influencing factors of treatment adherence of tuberculosis patients and development of rating scale. Social Medicine and Health Service Management of Huazhong University of Science and Technology. 2013.
